# ASAR: visual analysis of metagenomes in R

**DOI:** 10.1093/bioinformatics/btx775

**Published:** 2017-12-01

**Authors:** Askarbek N Orakov, Nazgul K Sakenova, Anatoly Sorokin, Igor I Goryanin

**Affiliations:** 1 Biological Systems Unit, Okinawa Institute of Science and Technology, Onna-son, Japan; 2 Department of Biology, School of Science and Technology, Nazarbayev University, Astana, Kazakhstan; 3 Mechanism of Cell Genome Functioning Laboratory, Institute of Cell Biophysics RAS, Pushchino, Russia; 4 Laboratory of Ion and Molecular Physics, Moscow Institute of Physics and Technology, Dolgoprundy, Moscow, Russia; 5 School of Informatics, University of Edinburgh, Edinburgh, UK; 6 Biodesign Centre, Tianjin Institute of Industrial Biotechnology, Tianjin, China

## Abstract

**Motivation:**

Functional and taxonomic analyses are critical steps in understanding interspecific interactions within microbial communities. Currently, such analyses are run separately, which complicates interpretation of results. Here we present the ASAR interactive tool for simultaneous analysis of metagenomic data in three dimensions: taxonomy, function, metagenome.

**Results:**

An interactive data analysis tool for selection, aggregation and visualization of metagenomic data is presented. Functional analysis with a SEED hierarchy and pathway diagram based on KEGG orthology based upon MG-RAST annotation results is available.

**Availability and implementation:**

Source code of the ASAR is accessible at GitHub (https://github.com/Askarbek-orakov/ASAR).

## 1 Introduction

Metagenomics allows investigation of microbial communities in a culture-independent way, taking advantage of the fact that estimated 99% of prokaryotes have not been successfully cultured (Schloss and Handelsman, 2005). In addition, the decreasing cost of sequencing and the increasing throughput of metagenomic data generation make the development of tools for functional, taxonomic and metabolic analyses of metagenomes extremely important (Hugenholtz and Tyson, 2008; Lindgreen *et al.*, 2016). However, even the most useful extant metagenomic analysis tools provide either only taxonomic (Menzel *et al.*, 2016) or only functional (Westbrook *et al.*, 2017) or both, but separately (Keegan *et al.*, 2016).

Although currently, annotations cannot be performed impeccably, crosslinking taxonomic and functional annotations at the read level could resolve many important questions, such as which taxonomic group in a sample is the main contributor to a particular function or metabolic pathway. Moreover, the capacity to analyze changes in microbiomes in the context of metabolic networks and to find the most interesting pathways, i.e. those most changed are the critical requirements for understanding biotechnological processes and would considerably improve analysis. These challenges have been addressed in ASAR (Advanced metagenomic Sequence Analysis in R). The core advantage of ASAR is its ability to perform taxonomic and functional analyses simultaneously, by subsetting and aggregating abundance data at various levels of taxonomic and functional hierarchies. It is designed to let researchers develop the most meaningful view of their data in a convenient way.

## 2 Materials and methods

The ASAR application was written in the R programming language (R Core Team, 2014) on the Shiny platform (Chang *et al.*, 2016). The application can both be used locally on machines with installed R or as a web-service.

Sequencing and annotation data are combined to form a 3D data cube (Kimball, 1996) with taxonomy, function and metagenome as dimensions. Interactively applying selection and aggregation operations at a user defined level, we provide an interactive interface for drill-down analysis of metagenomic data. For analysis, we combine the ‘best hit’ functional and taxonomic classification from SEED (Overbeek, 2005) and the ‘best hit’ functional classification from KEGG orthology (Kanehisa *et al.*, 2016) provided by MG-RAST (Keegan *et al.*, 2016). At the moment, we use the annotation files from MG-RAST, but any other annotation pipeline that assigns annotations at the read level could be incorporated as well. A detailed description of data preparation is available in the Supplementary Material.

## 3 Results

The ASAR interface consists of a main panel with seven tabs and a control panel. The control panel provides a set of parameter selectors to control the displayed tab content. When tabs share the same set of parameter inputs, these are maintained when moving across tabs to analyze different projections of the same data subset.

Users can select a color scheme for heatmaps from the RColorBrewer package (Neuwirth, 2014). All heatmaps and KEGG diagrams in the ASAR are downloadable as a high-resolution, publication quality images in PDF or PNG formats.

### 3.1 Three-dimensional dataset visualization

The combination of taxonomic and functional annotations in several metagenomic samples form a three-dimensional data cube, where each cell represents those reads mapped to a particular function and taxon in a particular metagenome. We have implemented the interactive tool for visual analysis of data cube contents by applying selection, aggregation and projection operations and by representing two-dimensional projections of selected subsets of the data cube as heatmaps. Taxonomic and functional dimensions are organized into a hierarchy, so the user can specify the level at which to select and aggregate data. All reads annotated with a chosen value at a selected level of a hierarchy are collected and aggregated according to their annotation at the aggregated level. In the metagenomic dimension, each metagenome is annotated with a set of user-defined metadata properties. So for this dimension, aggregation is implemented by averaging data that belongs to metagenomes with the same values at a selected property. The combination of operations described above allows precise selection of data together with concise and interpretable visualization.

#### *3.1.1* Function versus Taxonomy heatmap

This heatmap projects a data cube along the metagenomic dimension by combining functional and taxonomic data for single metagenome. This is useful for discovering the relationship between functions and taxonomic groups in a given metagenome.

#### *3.1.2* Function versus Metagenome heatmap

This heatmap projects a data cube along the taxonomic dimension by aggregating abundance data for a specified set of taxa. This is designed to compare abundances of functional groups in selected taxonomic groups between metagenomes.

#### *3.1.3* Taxonomy versus Metagenome heatmap

This heatmap projects a data cube along the functional dimension by aggregating the abundances of selected functional groups. It converges to standard taxonomic analysis if the root of the functional hierarchy is selected. This heatmap is designed for exploration of taxonomic groups that differ in abundance within selected metagenomes.

### 3.2 KEGG pathway abundance analysis

The KEGG Pathway Abundance heatmap shows pathways with genes that differ most in abundance for selected taxonomic groups and samples. The pathway diagram can be visualized in the KEGG Diagram tab with genes color coded by the pathview package (Luo and Brouwer, 2013). The color of each enzyme on the KEGG diagram represents the percentage of enzyme abundance provided by selected taxa in each metagenome. This allows estimation of the role of selected taxa in providing this function to the whole microbial community.

## Supplementary Material

Supplementary Data
