# Supplementary material for: ASAR: visual analysis of metagenomes in R
Source: Bioinformatics. 2017 Dec 1;34(8):1404–5. doi: 10.1093/bioinformatics/btx775 (PMC5905653; doi:10.1093/bioinformatics/btx775)
Supplement: Supplementary Data [file btx775_suppl_data.pdf]

Advanced metagenomic Sequence Analysis in R

Manual for version 1, revision 2

View on GitHub

Download .zip

Download .tar.gz

Askarbek Orakov, Nazgul Sakenova, Anatoly Sorokin and Igor Goryanin

Outline:

1. Summary
2. Introduction
3. ASAR application
4. Results
5. Parameters
6. Installation
7. Data Preparation
8. References

Summary

What is it? Functional and taxonomic analysis are critical in understanding interspecific interactions within microbial communities. Presently, these types of analysis are run separately, which makes results difficult to interpret. Here we present the ASAR interactive tool for simultaneous analysis of metagenomic data along three dimensions: taxonomy, function, and metagenome.

What is required of users? Users need to have R and some R packages installed in their computers. Additionally, terminal is needed in order to run the BASH script that will download, process, and save data in the appropriate format to be read by the application. Annotation files from MG-RAST are used as sample data. In order to use data from MG-RAST, only the project ID and user webkey must be supplied by the user.

Why should I use it? Advantages of the tool are 1) Integrated functional and taxonomic analysis; 2) Comparative analysis of KEGG pathway enrichments; 3) KEGG Pathway Maps; 4) User-friendly interface.

Introduction

For analysis of metagenome sequences by next-generation sequencing, short reads are mapped to the annotated sequence database. That kind of mapping provides information about the presence in a community of particular species, functional enzymes, and their abundances. So, for each community under consideration, we obtain a 2D matrix, where each row represents specific species and each column represents functions. Each cell of that matrix contains abundances of reads mapped to the particular function in particular species. The specific nature of that matrix is that both columns and rows form a hierarchy: a taxonomic tree for species composition and functional ontology/classification for protein functions.

It should also be mentioned, that we are rarely dealing with a single set of sequences from one community. Usually, at

least technical repeats and controls are present. Sets of samples forms the third dimension of our dataset (fig A). The community or samples direction usually does not have a hierarchical relationship between samples. Instead, it represents the structure of the design matrix, created to estimate the contribution of factors of interest to community composition and/or functions.

Figure A. **3D dataset**

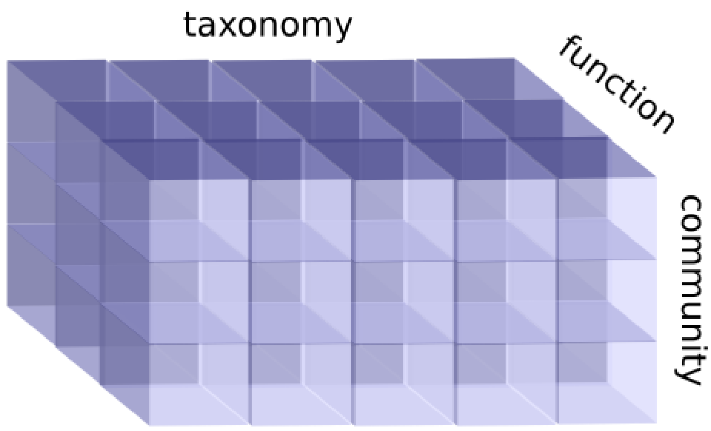

Datasets of dimensionality higher than 2 require special techniques for analysis and visualization, so in most modern applications, 3D datasets are reduced to 2D matrices by discarding one of their dimensions (fig B). For example, taxonomic analysis applications, like Kaiju [4], consider only samples and the taxonomy axis of the data cube, summing reads mapped to any function in a particular taxon into one cell. Similarly, functional analysis software, like Paladin [5], discards the taxonomic axis and analyzes only samples and function dimensions.

Figure B. **Projecting the 3D dataset along the function axis**

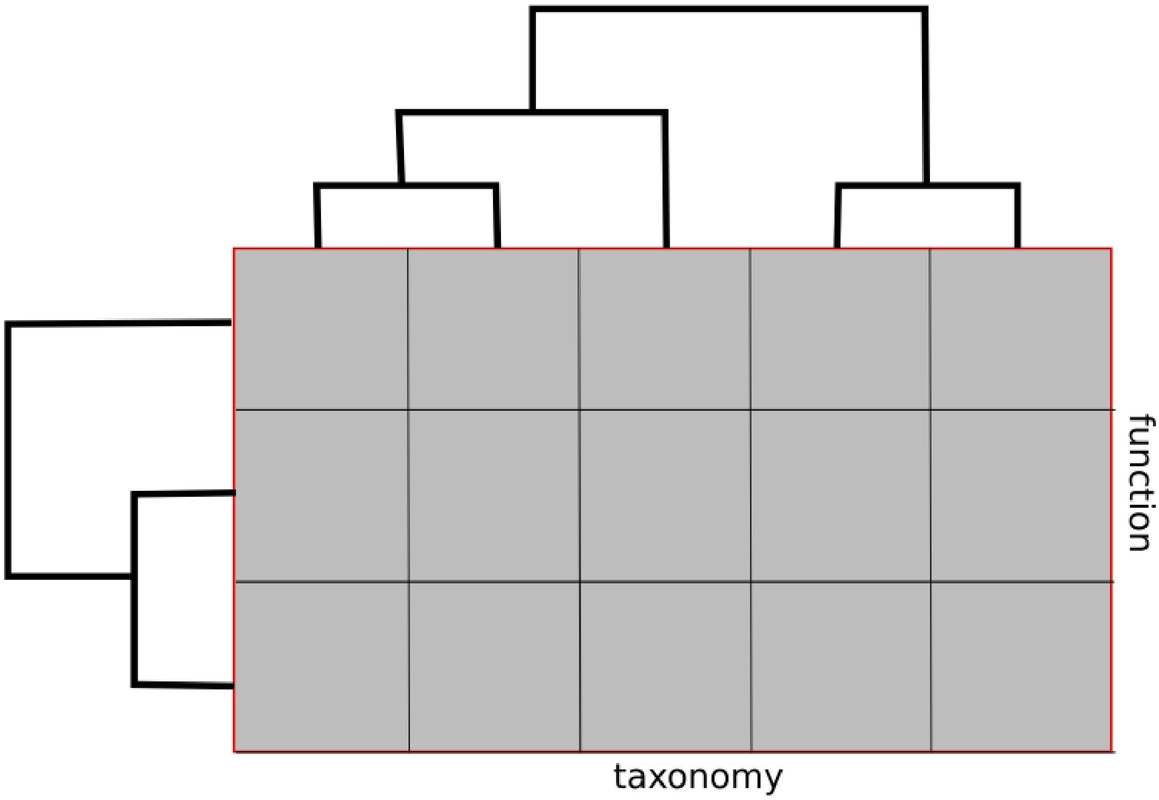

In ASAR we aim to provide dynamic visualization and an analysis framework to facilitate exploration of the whole 3D dataset. This type of analysis can provide valuable insights not only into the composition and functional abilities of a community, but also regarding the role of particular organisms in the community, the presence of symbiotic or antagonistic interactions between members of the community, and so on.

To fulfill this aim, we organize the dataset into a “data cube,” a concept developed in computer science in the mid 1980s to describe multidimensional datasets with complex relationships between the elements in each dimension (https://en.wikipedia.org/wiki/OLAP_cube). Data cubes are a common concept in business analytics and OLAP applications. To analyze the content of the data cube, a set of operations was defined: Slice, Dice, Roll-up, and Drill down. The Slice operation takes a specific value along one dimension and extracts the 2D subset corresponding to the selected value. In ASAR we have implemented the Slice operation for sample dimensions in the **Function vs. Taxonomy (F/T)** tab.

The Dice operation, when the user defines a set of values along different axes, is not implemented in ASAR explicitly, as we focused on Drill down and Roll-up operations.

The Drill down operation, which we called Selection (fig C), allows the user to navigate through the hierarchy by selecting an element at some higher level of the tree and to analyze the subset of the cube underneath that element. For example, the user can choose Deltaproteobacteria at the class level of taxonomy so as to restrict consideration to species and functions in that class only.

Figure C. **Selection operation**

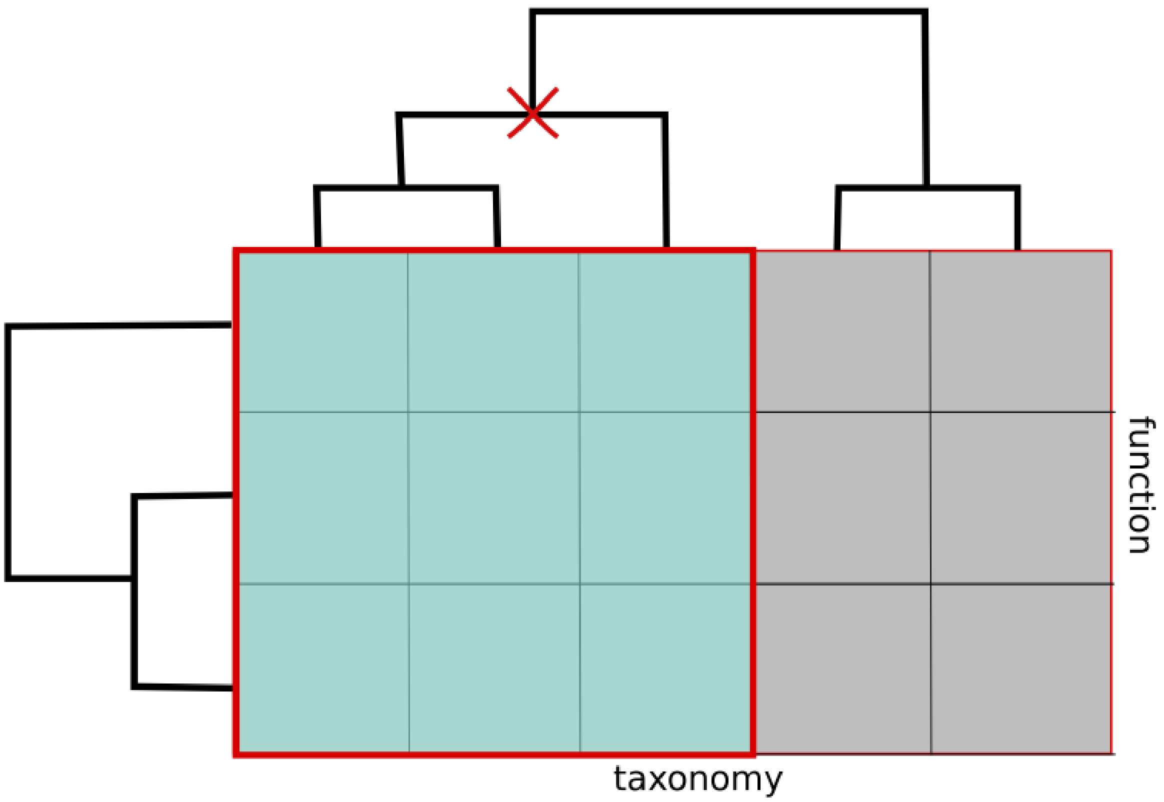

The Roll-up operation, which we called Aggregation, allows the user to summarize the data at some level of the hierarchy. For example, the reliability of data at the strain level is usually low, so it is common to Aggregate the data up to the genus level.

Figure D. **Aggregation operation**

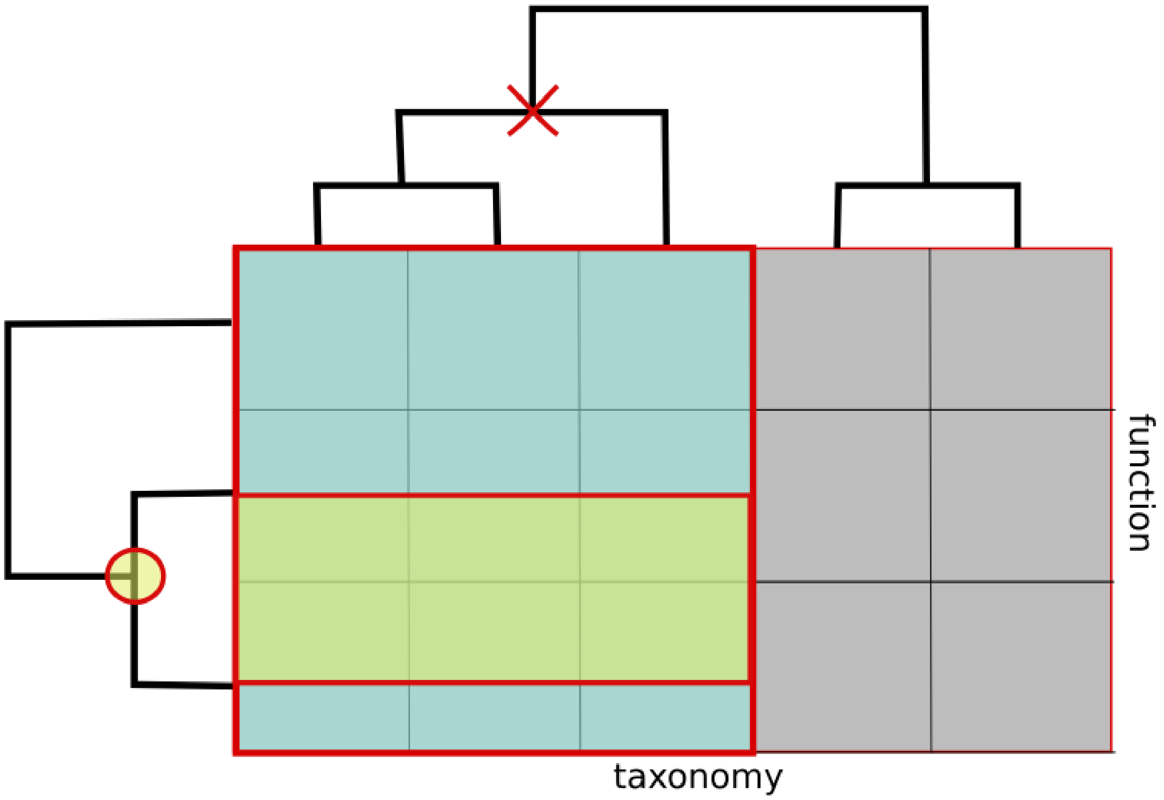

Despite having used taxonomy to illustrate the set of operations, all operations could be applied to the functional dimension, and with the caveat that samples do not form a tree structure, to the sample dimension.

ASAR application

The ASAR application implements two types of analyses. First, it builds 3D dataset with axes corresponding to taxonomy, functions, and metagenome samples [1,2]. Second, KEGG metabolic pathway analysis consists of a comparative analysis of pathway enrichment and visualization of pathways involved [3].

Since taxonomic and functional annotations have many groups at several levels and metagenome samples are numerous, two main data manipulations are implemented. First, "Selection" involves selecting one or more groups in each axis at specific levels in the function and taxonomy dimensions. Second, "Aggregation" involves selecting a level lower than "Selection" level at which selected data should be aggregated into groups of "Aggregation" level by summing read counts. "Aggregation" of metagenomes is done by averaging metagenomes with same defined name.

Sample data may be used to explore the app. To use your own data, please read the section, "Data Preparation."

Results

3D dataset (Function & Taxonomy & Metagenomes) Interactive Heatmaps

Heatmaps contain dendrogram trees to the left and above from the heatmap and column and row names below and to the right from the the heatmap. Dendrograms are generated with the "hclust" function in R and color key located up and to the left of heatmaps represents the distribution of colors in the heatmap. The value of a cell in the heatmap can be viewed by hovering the cursor above that cell. The value of the cell is the log2 value of the read count for that cell.

Figure 1. **Function vs Taxonomy (F/T)**

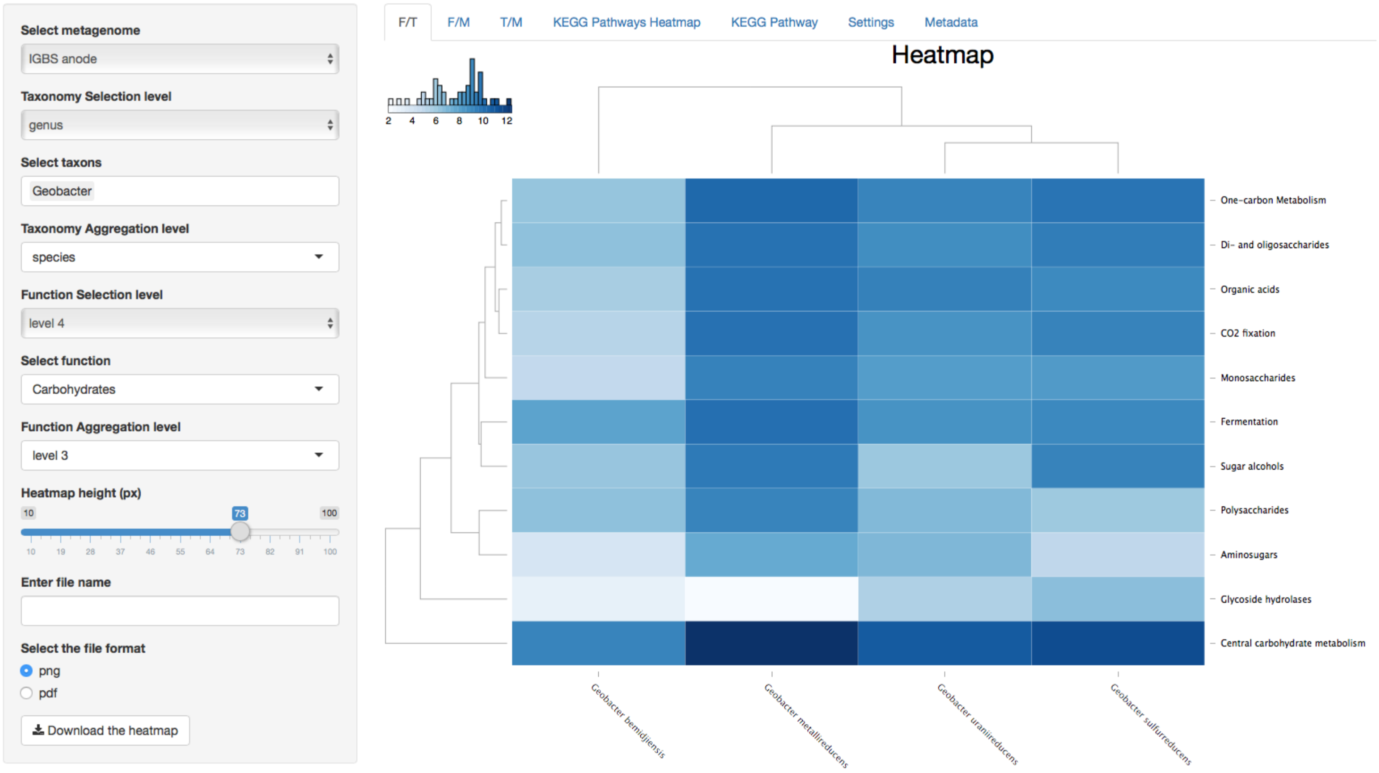

Figure 2. **Function vs Metagenomes (F/M)**

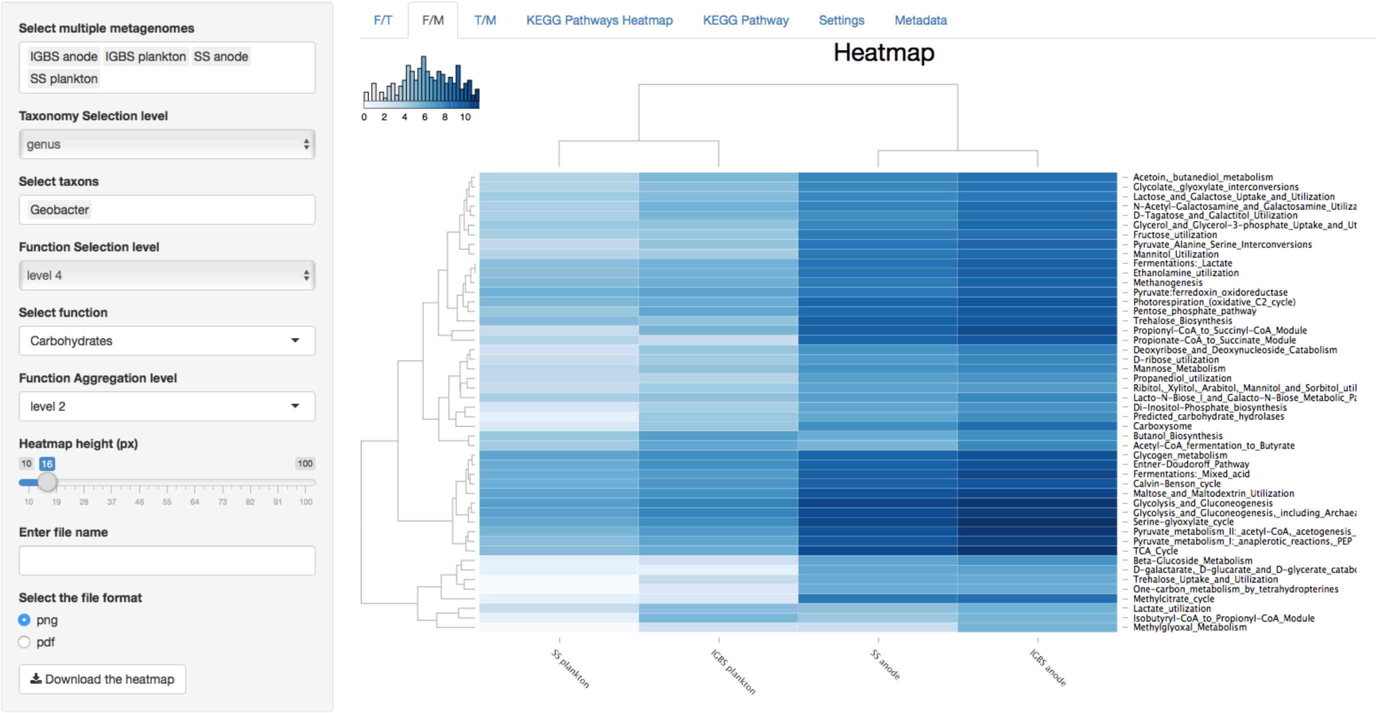

Figure 3. **Taxonomy vs Metagenomes (T/M)**

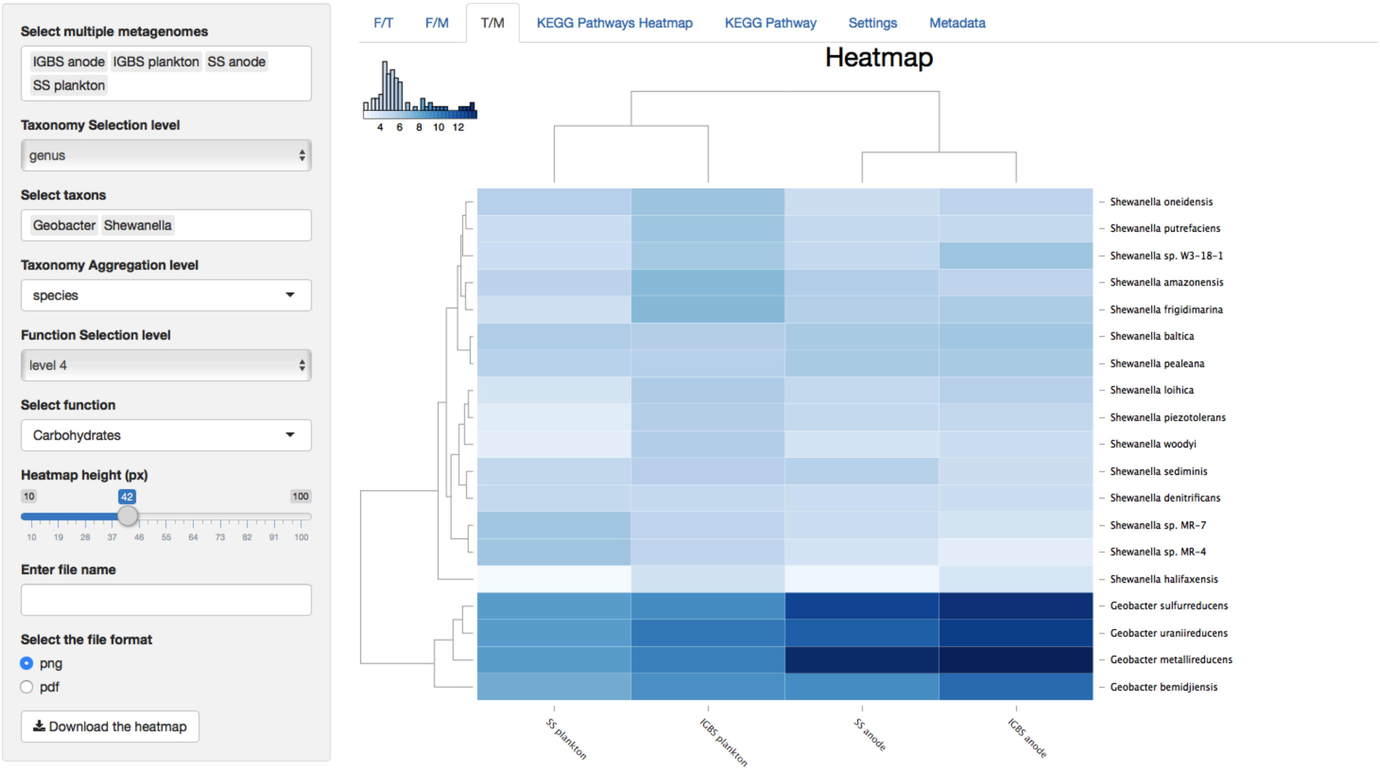

KEGG Pathways Heatmap

Figure 4. **KEGG Pathways Heatmap**

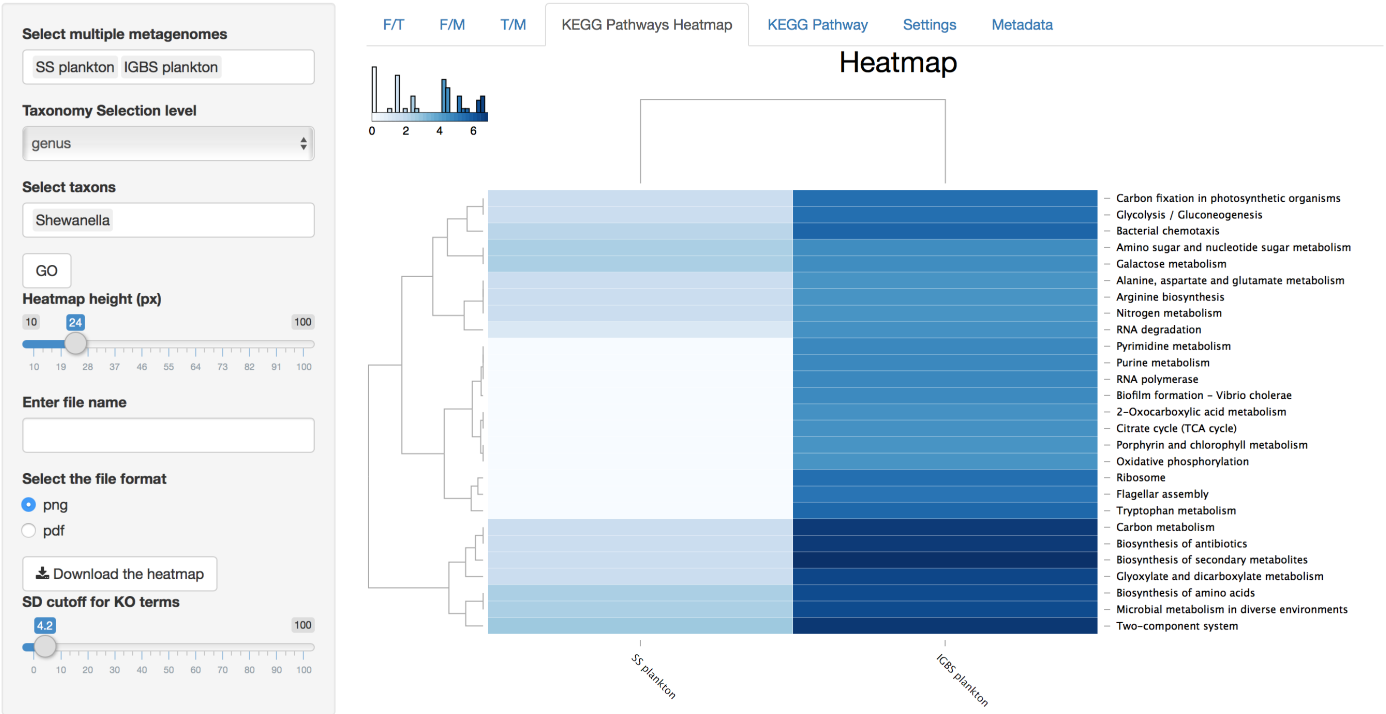

In addition to characteristics explained for 3D dataset heatmaps, KEGG Pathway Heatmap has a standard deviation cutoff explained in the parameters section for SD cutoff.

KEGG Pathway

Figure 5. **KEGG Pathway**

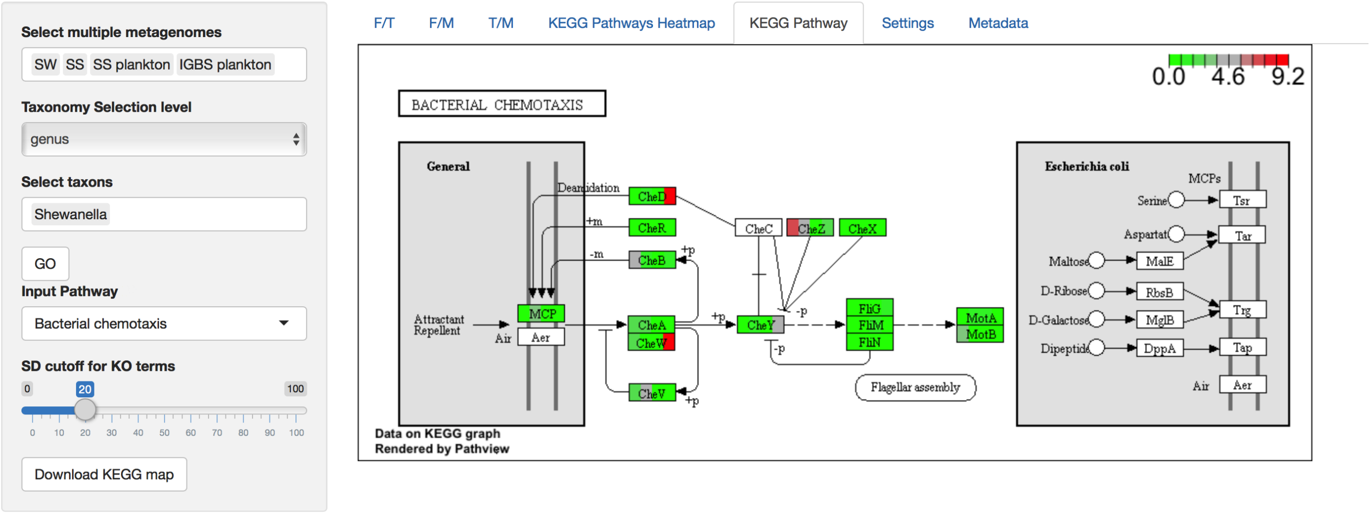

KEGG Pathway shows the metabolic pathway image for a selected KEGG pathway and color enzymes with known expression in metagenomes. Each rectangle representing an enzyme is horizontally divided into a number of selected metagenomes, where the coloration of partitions corresponds to values of selected metagenomes (i.e. Partitions are colored from left to right in the order of the selected metagenomes). Values of enrichment represent the percent contribution of a selected taxon from the total read count of the whole metagenome to this KEGG Orthology (enzyme). The color key represents a range from zero to a maximum value among KO's in the pathway.

Parameters

Metagenome Selection

The Function vs Taxonomy (F/T) heatmap requires selection of single metagenome, while all others allow selection of multiple metagenomes (see figure 6). Names of metagenomes shown for each selection can be changed by selecting a

specific column of metadata, rows of which will be used as metagenome names (see Figure 7). In order to consider several metagenomes as one, these metagenomes should be given the same name in the selected metadata column. In this case, abundances of reads from metagenomes with the same name are averaged in the new metagenome under a shared name before any abundance processing steps.

Figure 6. **Unlike other heatmaps, the F/T heatmap requires selection of single metagenome.**

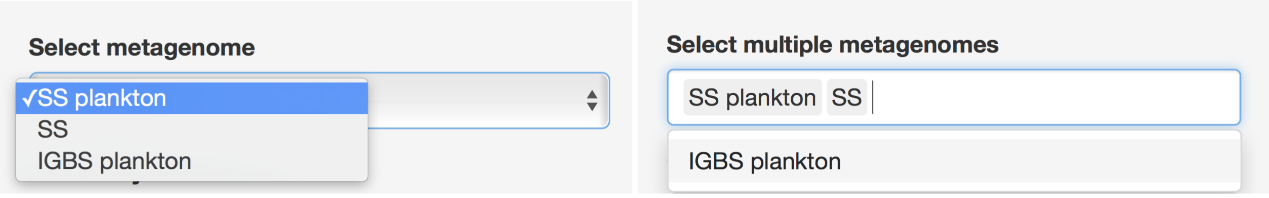

Figure 7. **Metagenomes' names selection to be displayed.**

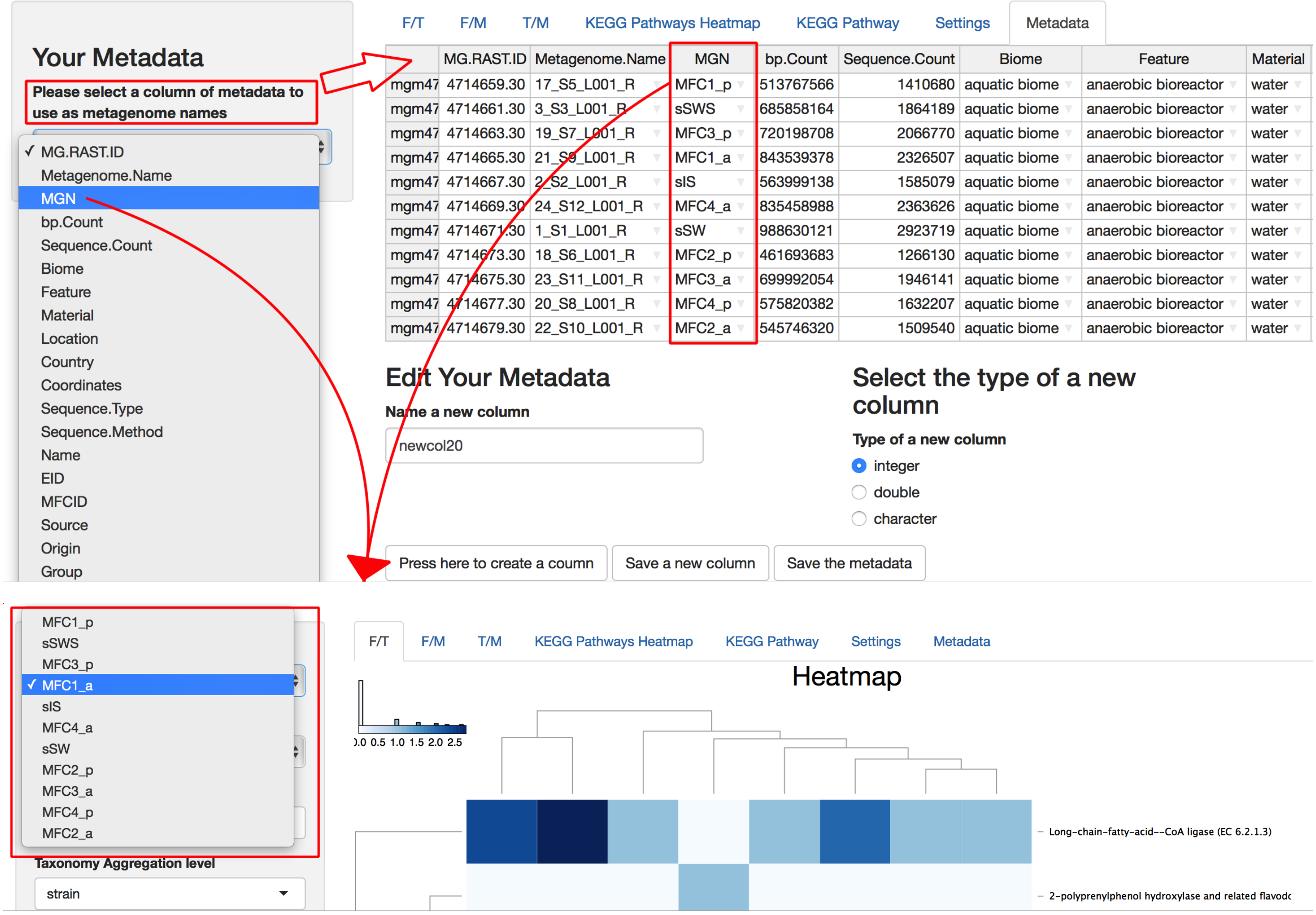

Taxonomy and Function Selection

There are 8 choices for taxonomy level, where "root" means all domains and 7 levels from "domain" to "strain." At all levels except "root," taxon selection gives a list of all available taxa at that level and multiple taxon selections are possible. At last, a second taxon level is used to aggregate selected taxa at that level. Functions are selected using the same principle except that Functions has 5 levels instead of 8 (see Figure 8).

Figure 8. **Taxonomy and Function Selection**

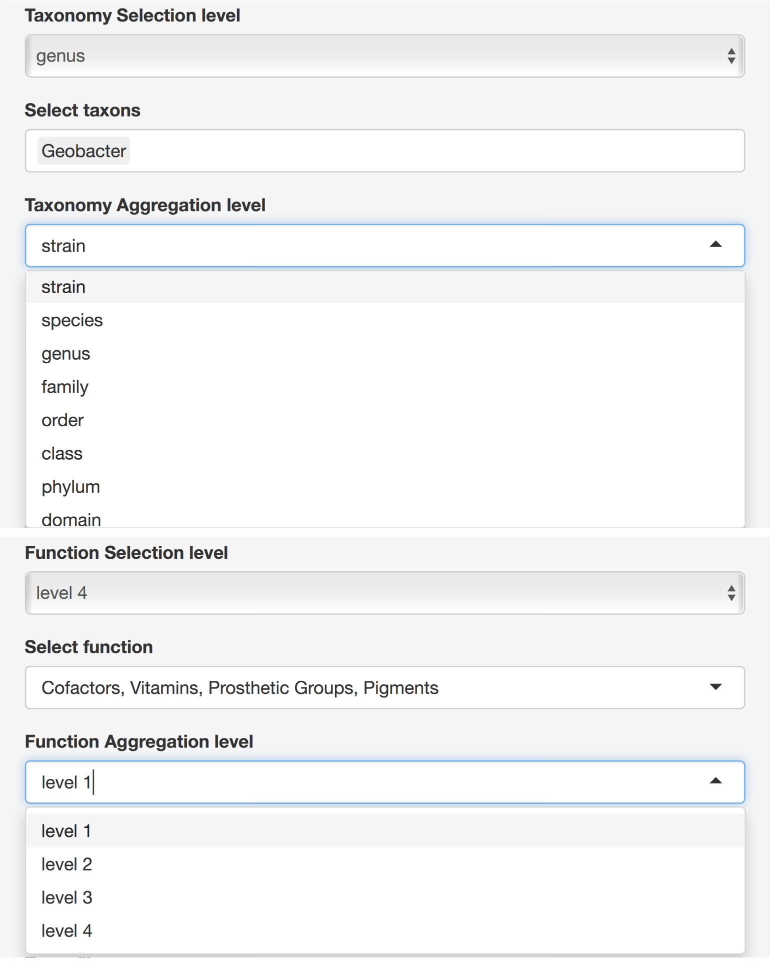

Pathway Selection for Building KEGG Pathway

The list of KEGG Pathways available for the current selection of metagenomes and taxa is displayed for the selection. Selecting Pathway will send the request to build a KEGG Pathway and may take up to several seconds, depending on the number of genes in the pathway.

Heatmap Height Selection

Default height of heatmaps is 20 pixels per row and is adjustable through a slider input parameter, which displays and sets the height of a single row (in pixels).

Image Download

Every heatmap can be downloaded by typing a user-defined file name in the "Enter file name" text input parameter, selecting image format (PNG or PDF), and subsequently pressing the Download button. The KEGG map image is downloaded in the same way, but without defining the file name. Alternatively, the map image can be saved by clicking the right mouse button (usual browser functionality), where the user can define the filename.

Standard deviation cutoff for KEGG Orthology terms

KEGG Pathway Heatmap and KEGG Pathway have an adjustable parameter called "SD cutoff for KO terms," which defines the standard deviation for individual KO's among all selected metagenomes. This value cuts off all KO's with SDs less than the value from Heatmap.

Parameters in Metadata Tab

Selection of column of metadata displays all columns of metadata and allows the use of rows in these columns as names of metagenomes in the metagenome selection parameter. Metadata values are editable and new columns with a name

specified by user can be added to the metadata. There are three types of new column that the user can select: "integer," "double," and "character." Pushing the "Save" button updates the default RData file with the current dataset. Pushing "Save a new column" button will update metadata, but current Rdata should be saved and run in a new session. When metagenome names from some column of metadata are used, identically named metagenome data will be averaged and analyzed as single metagenome. This is how aggregation of metagenomes is accomplished.

Parameters in Settings Tab

The Upload function can be used to upload Rdata files generated previously and the Save function can be used to save the current state of the loaded dataset. The Settings tab has entries for changing default values of functional and taxonomic levels. The palette of colors used for coloring heatmaps can also be selected. Pressing the "Save changes" button will save these default parameters for subsequent sessions.

Installation

R Packages from CRAN:

1. Package 'shiny' *version 1.0.3*
2. Package 'ggplot2' *version 2.2.1*
3. Package 'gplots' *version 3.0.1*
4. Package 'data.table' *version 1.10.4*
5. Package 'plyr' *version 1.8.4*
6. Package 'stringr' *version 1.2.0*
7. Package 'shinythemes' *version 1.1.1*
8. Package 'matrixStats' *version 0.52.2*
9. Package 'png' *version 0.1-7*
10. Package 'devtools' *version 1.13.2*
11. Package 'rhandsontable' *version 0.3.4.6*
12. Package 'RColorBrewer' *version 1.1-2*

Bioconductor:

1. Package 'pathview' version 1.14.0
2. Package 'biomformat' version 1.2.0
3. Package 'KEGGREST' version 1.14.1
4. Package 'limma' version 3.30.13

GitHub:

Package 'd3heatmap' by *"Alanocallaghan/d3heatmap"*

To run the app on your local machine:

1)Download RStudio/R

2) Install packages listed below.

```
install.packages("shiny",  dependencies = TRUE)
install.packages("devtools", dependencies = TRUE)
install.packages("ggplot2", dependencies = TRUE)
install.packages("gplots", dependencies = TRUE)
install.packages("RColorBrewer", dependencies = TRUE)
install.packages("data.table", dependencies = TRUE)
install.packages("plyr", dependencies = TRUE)
install.packages("stringr", dependencies = TRUE)
install.packages("shinythemes", dependencies = TRUE)
install.packages("matrixStats", dependencies = TRUE)
install.packages("png", dependencies = TRUE)
install.packages("rhandsontable", dependencies = TRUE)

##try http:// if https:// URLs are not supported
source("https://bioconductor.org/biocLite.R")
biocLite("pathview", suppressUpdates = TRUE)
biocLite("biomformat", suppressUpdates = TRUE)
biocLite("KEGGREST", suppressUpdates = TRUE)
biocLite("limma", suppressUpdates = TRUE)

library(devtools)
install_github("Alanocallaghan/d3heatmap")
```

3)To run the app user has to clone the repository to a local machine.

a. Open Terminal.

b. Change the current working directory to the location where you want the cloned directory to be made.

c. Run the command on Terminal:

```
git clone https://github.com/Askarbek-orakov/ASAR.git
```

d. The folder will be saved as “ASAR”. Open the folder and go to subfolder “R”, where you will find “app.R”. Please, open “app.R” with RStudio and then click on “Run App” button (ASAR/R/app.R).

Data Preparation

1) After cloning the repository as described above, in RStudio open ASAR/bash/prepareProject.Rmd .

2) Install packages listed below.

```
#Run this command in the console.

install.packages("pander",  dependencies = TRUE)
install.packages("knitr",  dependencies = TRUE)
install.packages("ggplot2",  dependencies = TRUE)
install.packages("plyr",  dependencies = TRUE)
install.packages("RJSONIO",  dependencies = TRUE)
install.packages("data.table",  dependencies = TRUE)
install.packages("RCurl",  dependencies = TRUE)
install.packages("xtable",  dependencies = TRUE)
install.packages("shiny",  dependencies = TRUE)

##try http:// if https:// URLs are not supported
source("https://bioconductor.org/biocLite.R")
biocLite("biomformat", suppressUpdates = TRUE)
```

3) Preparation of data from MG-RAST requires only the project ID and a webkey. Before running the code in

"prepareProject.Rmd" there should be two variables set for appropriate collection of the data:

webkey

```
#Run this command in the console.
 webkey <- "your_webkey_goes_here"
```

To have a webkey, user has to be registered in MG-RAST. To get your webkey in MG-RAST, press "show webkey," as indicated below.

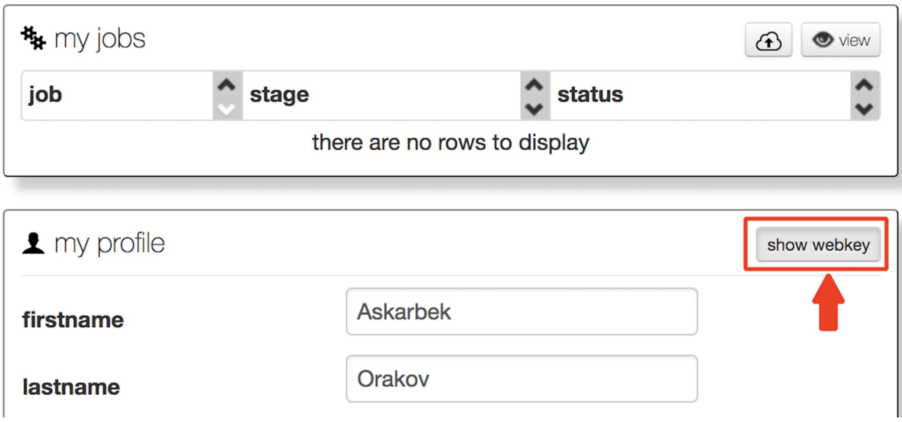

prjTMP

```
#Run this command in the console.
prjTMP <- "mgpXXXXX"
```

Project ID starts with "mgp". The example of Project ID is "mgp13644".

4) After packages have been installed and variables set, "prepareProject.Rmd" can be run.

5) In the same folder with this report you can find 'submit.sh' file which is required to fetch all data from MG-RAST server via API. To run the script on Terminal type

```
cd `r paste0('mkdir project.',proj.ID)`
./submit.sh
```

Once all jobs are finished run the *checkDownload.R*:

```
./checkDownload.R
```

If some files are missing or partially downloaded 'checkDownload.R' script will create 'resubmit..sh' script, which will reload missing files. If download is complete and functional the 'checkDownload.R' script will create Rdata file ready for use in ASAR.

The app can be used by

A. Exploring the pre-loaded example data set. This is a pre-loaded Metagome Sample taken from the swine waste example for exploring the app's features

B. Replacing initial 'pathview.RData' in in ASAR/R.

C. Uploading an .RData file containing your data, previously downloaded from the app session.

References:

[1] Keegan, K. P., Glass, E. M., & Meyer, F. (2016). MG-RAST, a metagenomics service for analysis of microbial community structure and function. Microbial Environmental Genomics (MEG), 207-233.

[2] Overbeek, R., Begley, T., Butler, R. M., Choudhuri, J. V., Chuang, H. Y., Cohoon, M., ... & Fonstein, M. (2005). The subsystems approach to genome annotation and its use in the project to annotate 1000 genomes. Nucleic acids research, 33(17), 5691-5702.

[3] Kanehisa, M., Sato, Y., Kawashima, M., Furumichi, M., & Tanabe, M. (2016). KEGG as a reference resource for gene and protein annotation. Nucleic acids research, 44(D1), D457-D462.

[4] Menzel, P. et al. (2016) Fast and sensitive taxonomic classification for metagenomics with Kaiju. Nat. Commun. 7:11257

[5] Westbrook, A., Ramsdell, J., Schuelke, T., Normington, L., Bergeron, R. D., Thomas, W. K., MacManes, M. D. (2017) PALADIN: protein alignment for functional profiling whole metagenome shotgun data. Bioinformatics, 33 (10), 1473–1478.

ASAR is maintained by Askarbek-orakov.
This page was generated by GitHub Pages.
